# Knowledge, Attitudes, and Practices (KAP) of the Relationship between Air Pollution and Children’s Respiratory Health in Shanghai, China

**DOI:** 10.3390/ijerph120201834

**Published:** 2015-02-05

**Authors:** Rui Wang, Yingying Yang, Renjie Chen, Haidong Kan, Jinyi Wu, Keran Wang, Jay E. Maddock, Yuanan Lu

**Affiliations:** 1 School of Public Health, Fudan University, 138 Yixueyuan Road, Xuhui District, Shanghai 200032, China; E-Mails: wang_r13@fudan.edu.cn (R.W.); yingyingyang13@fudan.edu.cn (Y.Y.); crj_1986@163.com (R.C.); kanh@fudan.edu.cn (H.K.); 12211020067@fudan.edu.cn (J.W.); 10301020044@fudan.edu.cn (K.W.); 2 Department of Public Health Sciences, University of Hawaii at Manoa, Honolulu, HI 96822, USA; E-Mail: jmaddock@hawaii.edu

**Keywords:** air pollution, children’s respiratory health, knowledge, attitudes, practices (KAP)

## Abstract

To assess the status of, and factors associated with, residents’ knowledge, attitudes, and practices (KAP) related to air pollution and respiratory health of children in Shanghai, we conducted a cross-sectional survey. Demographic factors associated with residents’ knowledge were identified by multiple logistic regressions. The questionnaires were completed by 972 participants, half from the Shanghai Children Hospital and the other half from the Jiading communities. Half of the participants’ scores of knowledge and attitudes were equal or greater than 8.0 on a 9-point scale, over 75% of respondents’ practice scores were equal to or less than 4.0. Our studies demonstrated a significant difference of average knowledge scores between the two groups (*t* = 1.27, *p* < 0.05). The parents’ educational level (OR = 1.89, 2.48) and average annual household income (AAHI) (OR = 2.37, 2.40, 2.12) were the two strongest factors on knowledge awareness. In addition, statistical analysis revealed a significant difference between the two groups in their attitudes towards air quality and their perception of the government’s efforts to alleviate it. The hospital and community groups also showed significant differences in practices geared towards protecting their children’s health. Nearly 90% of the respondents agreed that improving air quality is the responsibility of every citizen, and the joint action of governments and all citizens should be utilized for enhanced control. In addition, more resources should be allocated towards providing citizens with appropriate practices to help lessen the effects of poor air quality.

## 1. Introduction

Clean air is considered a basic tenet of human health and well-being. As the largest developing country in the world, China has started to experience economic prosperity across many sectors. However, rapid economic growth has been accompanied with sharp increases in environmental pollution, most notably, air pollution. Energy consumption is the main source of air pollutants in China, including: fine and course particulate matter (PM_2.5_ and PM_10_), sulfur dioxide (SO_2_), nitric oxide (NO*_x_*), and carbon monoxide (CO) [1]. In 2012, China issued updated Standards for Ambient Air to control air pollution [2]. Following those updates, the China Environmental Aspect Bulletin reported that only three cities met the air quality standards, while 95.9% of the cities exceeded them in 2013 [3]. According to the 2013 Shanghai Environment Bulletin, there were 124 polluted days out of the 365 (approximately 34%) measured by Air Quality Index (AQI) in Shanghai. The main contaminant was PM_2.5_ with an annual average daily value of 62 μg/m^3^, which is higher than the reported 27 μg/m^3^ in Class II (standards for residential areas) cities in China [4]. As a major atmospheric pollutant in China, PM_2.5_ has led to more than 1.2 million premature deaths and 25 million years of life lost in 2010 [5]. PM_10_ pollution is also an issue, and substantial health loss was observed in 113 major Chinese cities due to PM_10_ in 2006. This included 299,700 premature deaths, 92,699 cases of chronic bronchitis, and 7,625,100 outpatient cases [6]. It was estimated that the total economic health impact cost of poor air quality was approximately RMB 341.40 billion (approximately USD 55.08 billion), 87.79% of which was attributable to premature deaths [7]. 

Air pollution is known to lead to global climate changes. There is variety of air pollutants including greenhouse gases, but their effect to the climate changes can be very differently. The current amount of greenhouse gases, the natural portion of our atmosphere, has dramatically increased due to human activities, including increased car exhaust and the release of pollutants from coal-based power plants and varied manufacture factories. Carbon dioxide is well-known greenhouse gas and a common part of the car exhaust can trap heat from sun in the atmosphere, leading global warming and climate changes. More global warming and climate changes are expected to occur in future centuries if this issue cannot be seriously considered and addressed properly today. Global warming/climate changes may cause many health problems, including extreme heat, natural disasters (*i.e.*, drought), spreading of vector-borne diseases, allergens, and very poor air quality, which may lead to various respiratory health problems, particularly to children. Recent researches have shown that maternal exposure to air pollution during pregnancy could lead to early fetal loss [8], premature delivery [9] and lower birth weight [10]. Postnatal children are particularly affected by poor air quality as their immune system and lung functions are not fully developed, and they also spend more time outside. Air pollution could change children’s pulmonary health and decline lung function, causing asthma, pneumonia, chronic cough and bronchitis symptoms [11]. In a 2010 investigation of Shanghai children and teenagers’ respiratory diseases, the prevalence of respiratory illness was shown to have increased since 1988, with a prevalence of 1.50%, 3.34%, and 4.50%, respectively, in 1988, 2000 and 2009 [12]. Moreover, some studies concluded that some 346,000 respiratory hospital admissions could be avoided if urban air quality met Class II standards of AQI [13]. This illustrates that air pollution remains an important health problem in China, and will continue to seriously affect people’s health if there is not significant improvement in the future. 

A “Knowledge, Attitudes, and Practices (KAP)” survey is a representative study of a specific population that aims to collect data on what is known, believed and done in relation to a particular topic [14]. We conducted this survey to get a better understanding of residents’ knowledge of current air pollution, their attitudes toward air pollution control, and practices of protection in Shanghai. In addition, two study sites were selected to comparatively determine if there is a difference of KAP between respondents whose children were relatively healthy (community group) or unhealthy (hospital group). Since there are very few reports about residents’ KAP of air pollution and children’s respiratory health in China, this study is directed to exploring this issue by using Shanghai as an example, particularly to understand public’s knowledge, attitudes, and practices related to current air pollution and respiratory health of children in China, and what are the associated factors affecting with their KAP. Such information could be vitally important for local government for the development of more impactful policies and effective measurements to control air pollution and improve air quality in future.

## 2. Materials and Methods

### 2.1. Study Design and Sample

We conducted a cross-sectional survey aimed at investigating KAP of caregivers in Shanghai, China related to air quality. We selected two different groups as survey subjects to compare the knowledge differences between the respondents of relatively unhealthy children and the respondents of the healthy children. We selected the Shanghai Children Hospital as a survey site since it is the largest children’s hospital located in the Minhang District. The other survey site, Jiading, was randomly sampled from eighteen districts in the urban area of Shanghai. According to the design, residents who were 18 years or older, and were caregivers of a child between 1 and 12 years old of age were eligible for the study. Those who qualified were briefly told about our study and asked if they were willing to participate. 1000 subjects were interviewed.

### 2.2. Data

A 45-item questionnaire designed by public health experts was used in this study. The survey was conducted in a face-to-face interview style, which took about 10–15 min to complete. The questionnaire consisted of five parts:

#### Part 1: Social Demographics

There were nine questions in Part 1 that inquired about general information such as: the children’s age, gender, the patients’ or caregivers’ age, gender, educational level, place of residence, as well as average annual household income (AAHI).

#### Part 2: Information about Air Pollution and Children’s Respiratory Health

There were nine questions in Part 2 inquiring about air pollution and children’s respiratory health, such as general perceptions on air quality, children’s health status and respiratory diseases, *etc*.

#### Part 3: Knowledge Related to the Local Air Quality and the Correlation between Air Pollution and Children’s Respiratory Diseases

There were nine questions in Part 3 that inquired about residents’ knowledge of local air quality and how it related to children’s respiratory diseases. A correct answer was given a score of 1 and an incorrect answer was scored with a 0. The scores varied from 0–9 points and were classified into “high knowledge” levels of scores higher than 5.4 (60% of participants obtained the highest score of 9) and scores lower than 5.4 were classified into “low knowledge” levels. We defined the knowledge awareness rate as the percentage of respondents who were classified into “high knowledge” levels.

#### Part 4: Attitudes Regarding the Government’s Policies and Measures of Improving Air Quality

There were nine questions in Part 4 that examined the attitudes of the residents towards governmental policies and measures of improving air quality. A positive answer was given a score of 1 and a negative answer was given a score of 0. The scores were varied from 0–9 points.

#### Part 5: Practices of Protecting Children’s Respiratory Health

There were nine questions aimed at understanding the general practices of the residents in terms of polluted air self-protection, including: avoiding going outside, wearing masks, keeping the windows and doors closed, etc. A positive answer was given a score of 1 and a negative answer was given a score of 0. The scores varied from 0–9 points. The scores were classified into three levels (good practice 7.3–9.0 points, fair practice 4.6–7.2 points, and poor practice 0–4.5 points). The frequency of good practice levels ranged from 81%–100%. The frequency of fair practice levels ranged from 51%–80%, and the frequency of poor practice levels was 50% or less.

### 2.3. Quality Control

A literature review indicated that only a few studies focused on parents’ or caregivers’ attitudes and perceptions towards air pollution in China. Therefore, the questionnaire was designed for the specific needs of this study. Fudan University public health graduate students implemented and conducted the survey. Surveys were extensively pre-tested prior to study implementation to ensure that potential participants understood questionnaires well in Minhang and Xuhui Districts in Shanghai. A total of 78 questionnaires were completed, including 47 questionnaires from the Shanghai Children Hospital in Minhang and 31 questionnaires from the Xuhui communities. Investigators and data collectors participated in daily debriefing meetings post-survey collection to examine the data and discussed ways to improve the interview protocols.

### 2.4. Data Analysis

Completed questionnaires were imported into a computer using Epidata 3.1. Excel 2010 was applied to manage data. The Statistical Package for the Social Sciences (SPSS) version 20.0 was used for data analysis. The alpha level was set at 0.05 to determine statistical significance. Five independent variables, including: (1) respondents’ gender, (2) respondents’ age, (3) respondents’ educational level, (4) place of residence, (5) AAHI and (6) the child’s health status, were statistically analyzed using chi-square test. 

A non-conditional multiple logistic regression model was used to identify possible factors (respondents’ age, respondents’ educational level, AAHI, and the child’s health status) associated with residents’ knowledge related to the local air quality and the correlation between the air pollution and children’s respiratory diseases. The results were presented as an odds ratio (OR) value with a 95% confidence interval (95%CI). Spearman’s Rank Correlation Coefficient was used to describe the strength and direction of the relationship among knowledge, attitudes and practices (KAP).

### 2.5. Ethical Considerations

The institutional Review Board of Human Studies Program at the University of Hawai’i at Manoa approved this study. Local institution protocols at Fudan University were also followed to protect participants’ confidentiality.

## 3. Results

General demographic characteristics are summarized in Table 1. The study was conducted on a total of 1000 subjects, 972 questionnaires were completed (response rate 97.2%), including 478 of 500 respondents in the Shanghai Children Hospital (response rate 95.6%) and 494 of 500 participants in the Jiading communities (response rate 98.8%). Of the 972 subjects, 49.1% were males and 50.9% were females; respondents’ mean age was 33.7 (ranging from 21 to 66) years old with a standard deviation of 7.6. About three-fifth respondents had at least a high school education, although the portion of respondents with at least a high school education was higher in the hospital setting (83.6%) than in the community (41.3%). Most respondents’ AAHI was greater than RMB 20,000; only 7.7% of respondents’ reported an AAHI less than RMB 20,000 in the hospital setting, while 22.2% of respondents reported less than RMB 20,000 per year in the community setting. According to our results, only a few participants thought their children were unhealthy, 11.0% in the hospital setting and 2.5% in the community setting, respectively.

Overall, 80.5% of the study respondents were classified into “high knowledge” levels, whose scores were above 5.4 (82.2% in the hospital setting, 78.9% in the community setting). To prevent subjective judgment, we compared the different knowledge scores between the total subjects of two groups (7.32 in the hospital setting, 7.01 in the community setting), t-test showed statically significance (*t* = 1.27, *p* < 0.05). 

**Table 1:** General demographic information of the interviewees.

Demographic Factors	Hospital		Community		Total	
	*n*	%	*n*	%	*n*	%
Respondents’ Gender						
Males	208	43.5	267	54.1	475	48.9
Females	270	56.5	227	45.9	497	51.1
Respondents’ Age						
18–29 years	110	23.0	81	16.4	191	19.6
30–39 years	316	66.2	308	62.4	624	64.2
≥40 years	52	10.8	105	21.2	157	16.2
Respondents’ Educational Level						
≤Junior High School	79	16.4	290	58.7	369	38.0
High School-College	197	41.3	145	29.3	342	35.2
≥College	202	42.3	59	12.0	261	26.8
Place of residence						
City	124	26.0	122	24.7	246	25.3
Countryside	354	74.0	372	75.3	726	74.7
AAHI^*^ (RMB)						
＜20,000	37	7.7	110	22.2	147	15.1
20,000–50,000	98	20.6	180	36.4	278	28.6
50,000–100,000	149	31.2	133	26.9	282	29.0
＞100,000	194	40.5	71	14.5	265	27.3
Child’s Health Status						
Healthy	209	43.8	350	70.9	559	57.5
Fair	216	45.2	132	26.6	348	35.8
Ill	53	11.0	12	2.5	65	6.7

^*^ AAHI = Average annual household income.

Results (Table 2) of chi-square tests showed that the two factors most affecting the knowledge level of respondents were parents’ educational level (both of the hospital setting and the community setting, *p* < 0.05) and AAHI (in the community setting, *p* < 0.05). Moreover, according to the non-conditional multivariate logistic regression analysis results (Table 3), respondents’ educational level and AAHI were also the two strongest statistically significant factors on the knowledge scale (percentage of “high knowledge”). Compared with the reference group (≤ Junior high school education), the knowledge of respondents with a high school diploma or an undergraduate degree, and respondents with more than an undergraduate degree were higher, with OR values of 1.89 (95%CI 1.10–3.25) and 2.48 (95%CI 1.18–5.71), respectively (*p* < 0.05). Taking “AAHI＜RMB 20,000”as the control group, the knowledge awareness rate of RMB 20,000–50,000, 50,000–100,000 and ＞100,000 per year groups were higher, the OR values being 2.37 (95%CI 1.28–4.38), 2.40 (95%CI 1.24–4.65) and 2.12 (95%CI 1.04–4.64), respectively (*p* < 0.05). Respondents’ knowledge levels increased with their educational levels. Meanwhile, the respondents’ knowledge levels also increased with a higher AAHI. 

**Table 2:** Respondents’ general awareness rate of knowledge towards air pollution to children’s respiratory health.

Demographic Factors	Hospital		Community		Total	
	*χ*^2^	*p-*value	*χ*^2^	*p-*value	*χ*^2^	*p-*value
Respondents’ Gender	0.18	>0.05	0.16	>0.05	0.138	>0.05
Respondents’ Age	4.24	>0.05	4.57	>0.05	4.14	>0.05
Respondents’ Educational Level	21.92	<0.05	11.19	<0.05	33.00	<0.05
Place of residence	0.48	>0.05	1.90	>0.05	2.18	>0.05
AAHI (RMB)	2.43	>0.05	11.02	<0.05	10.50	<0.05
Child’s Health Status	0.46	>0.05	4.87	>0.05	4.40	>0.05

**Table 3:** Non-conditional multivariate logistic regression analysis for the association between socio-demographic and air pollution knowledge levels.

Variable(s)	β	OR	OR 95%CI ^*^	*p*-value
Respondents’ age (age ≥ 40 control group)				
18–29 years	0.35	1.42	0.65~3.10	＞0.05
30–39 years	0.01	1.01	0.56~1.83	＞0.05
Respondents’ Educational Level (≤Junior high school control group)				
Middle (High School-College)	0.64	1.89	1.10~3.25	＜0.05
High (≥College)	0.91	2.48	1.18~5.17	＜0.05
AAHI (RMB) (income ＜ 20,000 control group)				
20,000–50,000	0.86	2.37	1.28~4.38	＜0.05
50,000–100,000	0.87	2.40	1.24~4.65	＜0.05
＞100,000	0.79	2.12	1.04~4.64	＜0.05
Child’s Health Status (Ill control group）				
Healthy	0.21	1.23	0.50~3.03	＞0.05
Fair	0.81	2.26	0.86~5.90	＞0.05

^*^ CI = confidence interval; OR = odds ratio. Note: assigned “the score of knowledge ≥ 5.4” to 1, others to 0; Assigned “age ≥ 40” to 0 (control group), “18–29” = 1, “30–39” = 2; Assigned “parents educational level ≤ Junior high school” to 0(control group), “High school and College” = 1, “≥Undergraduate school” = 2; Assigned “income＜RMB 20,000” to 0(control group), “RMB 20,000–50,000” = 1, “RMB 50,000–100,000” = 2, “＞RMB 100,000” = 3; Assigned “Ill” to 0(control group), “Healthy” = 1, “Fair” = 2.

Attitudes of the residents towards government policies and actions concerning air pollution in Shanghai are shown in Table 4. 54.3% of respondents in the hospital setting believed that the air quality in Shanghai was “poor” or “very poor” last year (2013), only 27.4% of the interviewees from the community had the same opinions. The Chi-Square test between the answers towards 2013 Shanghai air quality between the hospital and community groups was statically significant (*χ*^2^ = 97.85, *p* < 0.05). In addition, 75.3% of respondents from the hospital perceived that the air quality was “much worse” or “a little bit worse” compared with 5 years ago, while only 52.3% from community group consider today’s air quality is not as good as five years ago. The answers towards air quality compared 5 years ago between the two groups were also significantly different (*χ*^2^ = 98.44,* p* < 0.05). About 60.9% of respondents in the hospital thought the Shanghai government did not spend enough money for preserving and protecting the local environment, while only 34.7% of interviewees in the community setting thought the government had spent an inadequate amount. 29.9% of community respondents thought that the government spent an adequate amount of money for preserving and protecting the environment; the percentage in the hospital respondents was only 7.5% (*χ*^2^ = 103.99, *p* < 0.05). Almost a third of respondents from both groups expressed no knowledge in regards to the resources government spent on air quality improvement. Around 90% of the subjects (89.1% in the hospital setting, 89.6% in the community setting) agreed that improving the air quality is the responsibility of every citizen.

**Table 4:** Residents’ attitudes to the government policies.

**How do you rate the overall air quality in shanghai last year?**											
**Group**	**Very Good**		**Good**		**Fair**	**Poor**			**Very Poor**		**Don’t Know**
Hospital	2.1%		4.8%		36.5%	33.6%			20.7%		2.3%
Community	8.3%		16.2%		46.0%	18.5%			8.9%		2.1%
Total	5.2%		10.6%		41.4%	25.9%			14.7%		2.2%
**How do you rate the air quality in Shanghai last year compared to 5 years ago?**											
**Group**	**Much Better**		**A Little Better**		**No Difference**	**A Little Worse**			**Much Worse**		**Don’t Know(Live in＜5 Years)**
Hospital	1.5%		7.3%		8.4%	34.2%			41.1%		7.5%
Community	13.4%		15.6%		11.4%	32.3%			20.0%		7.3%
Total	7.5%		11.5%		9.9%	33.2%			30.5%		7.4%
**How do you feel about the amount of resources Shanghai government spends for preserving and protecting the local environment?**											
**Group**		**Too Much**			**Much**	**Right Amount**	**Not Quite Enough**	**Too Little**		**Don’t Know**	
Hospital		0.8%			5.2%	1.5%	41.1%	19.8%		31.6%	
Community		3.9%			14.2%	11.8%	24.5%	10.3%		35.3%	
Total		2.4%			9.8%	6.7%	32.7%	15.0%		33.4%	
**Do you agree that improving the environment is the responsibility of every citizen?**											
**Group**		**Strongly Agree**		**Agree**		**Disagree**	**Strongly Disagree**	**Don’t Know**			
Hospital		55.1%		34.0%		2.3%	0.2%	8.4%			
Community		45.6%		44.0%		6.3%	1.0%	3.1%			
Total		50.3%		39.1%		4.3%	0.6%	5.7%			

Table 5 illustrates the respondents that answered a total of 9 questions with the total score of 9. Evaluating the distribution of respondents in the hospital, the community, and as a whole, there were 0.4%, 0.0% and 0.2% of respondents who scored in the “good practice” range. About 15% of respondents scored in the “fair practice” range (18.2% in the hospital setting, 13.2% in the community setting), while most of our subjects’ scores were distributed in the “poor practice” range. The average practice scores for all respondents in the hospital, the community, and as a whole, were 3.13, 2.95, and 3.04 out of 9 points, respectively. The results of fisher exact test showed that significant difference existed between the two groups (*χ*^2^ = 6.50, *p* < 0.05).

A Spearman rank correlation was used to evaluate the possible association between knowledge-attitudes (K-A), knowledge-practices (K-P), and attitudes-practices (A-P) scores in the total group. There were low positive correlations between K-A, K-P and A-P (Spearman rank correlation coefficients were 0.40, 0.37 and 0.31 respectively, *p* < 0.01), as shown in Table 6. The Spearman rank correlations of the two groups were closed to the total results, which were not shown in the table.

Cronbach’s alpha coefficient, as the measure of internal reliability of our questionnaire, was 0.86. A Kaiser-Meyer-Olkin value of 0.90 and a Bartlett spherical test of 9449.77 (*p* < 0.001) in an exploratory factor analysis of the 27 entries showed that the factor analysis was feasible. The method extracted 8 factors where the eigenvalues were greater than 1 after the Varimax orthogonal rotation, cumulative contribution rates were 62.33% of the variance. These tests of questionnaire indicated sufficient evidence for reliability and construct validity.

**Table 5:** Distribution of practices towards protecting children’s respiratory health.

Practices Levels	Good Practice (81%–100%)	Fair Practice (50%–80%)	Poor Practice (Less than 50%)
Hospital	0.4%	18.2%	82.4%
Community	0.0%	13.2%	86.8%
Total	0.2%	15.6%	84.2%

**Table 6:** Correlations between knowledge, attitudes, and practices in total respondents.

Variable(s)	Knowledge	Attitudes	Practices
Knowledge	1	--	--
Attitudes	0.40 ^**^	1	--
Practices	0.37 ^**^	0.31 ^**^	1

^**^ Correlation is significant at the *p* < 0.01 level (2-tailed).

## 4. Discussion

Air pollution has detrimental effects on all who are exposed, especially children who are most vulnerable to poor air. Today, air pollution has become a major environmental concern in China and the world. China’s economic development has brought along with a series of complex social and environmental issues. Our study attempts to evaluate Shanghai residents’ KAP of air pollution as it relates to children’s respiratory health. We aim to utilize this information to impact Chinese health policies to improve the situation of poor air quality plaguing all of China, especially its urban areas.

Our results have shown that approximately 80% of respondents were classified into the “high knowledge” levels, having basic knowledge about air pollution, children’s respiratory health, and the correlation between them. It is promising that more and more people have begun to focus on the negative impacts of air pollution, particularly that affecting children. Indeed, there is growing evidence that the fetus and young children are especially susceptible to particulate matter [15], polycyclic aromatic hydrocarbons, nitrosamines, pesticides, lead, mercury, polychlorinated biphenyls, and environmental tobacco smoke [16]. Exposure to these toxicants may be related to the increases of childhood asthma, cancer, and developmental disabilities in recent decades [17,18,19,20,21]. Long-term exposure to higher levels of outdoor air pollution was negatively associated with cardiorespiratory fitness, especially in Chinese schoolchildren [22,23]. Young children remain particularly susceptible to developmental toxicants for several years after birth because the brain continues to develop with the growth of glial cells and myelination of axons in the postnatal period [24,25]. The increased vulnerability of fetuses and children to environmental pollutants stems from their greater absorption, slower clearance of toxicants and decreased ability to detoxify exogenous chemicals or to repair damaged DNA [26,27], since lung development continues through a child’s sixth to eighth year of life [28]. 

According to our non-conditional multivariate logistic regression analysis, respondents’ educational level and AAHI were positively correlated with the knowledge awareness rate of air pollution and children’s respiratory health, indicating that better educated or relatively affluent residents were more concerned about air quality. For the better-educated respondents, in our study those who were more educated were more likely to recognize the negative health implications stemming from air pollution exposure; the greater awareness rate of knowledge associated with exposure is consistent with previous studies demonstrating that individuals with higher education have more knowledge and easy access to the information about air quality [29]. Better-educated individuals are more likely to express concerns about air pollution, which means they are more sensitive to the change of air quality at the same time. Results of Badland’s research also showed that higher educated people were more likely to recognize air pollution harmed their health when compared to referent categories [30]. Interestingly, research from the United Kingdom indicated the least deprived neighborhoods (which can be used as a proxy measure of higher education attainment) objectively recorded lower air pollution concentrations when compared to highly deprived communities [31]. Further research needs to investigate how the interaction between educational-level and awareness of air pollution. 

As for the AAHI correlation with air quality knowledge, those who have higher household income are more concerned about air quality and care about children’s respiratory health. The lower income respondents may be more likely to focus more attention on making ends meet, rather than improving their quality of life. It is difficult for low-income individuals to spend a lot of time learning the indirect health effects of environmental pollution on children. Previous studies also found a positive relationship between income and environmental concern, suggesting that the poorer tend to be less concerned about air quality [32,33]. According to Lawrence’s study, children from relatively low-income families have greater risk for poor health outcomes than middle-class and affluent children [34]. Mukherjee sampled 1724 residents of Calcutta and found that respondents with lower socioeconomic status had less awareness and concerns in response to air pollution [35]. All these findings are in accordance with results of our study. To date, most of the research has focused on links between income inequality and adverse health effects [36], while few studies focus on the correlation between income and knowledge of air pollution effects.

However the respondents’ practices scores were much lower than their knowledge and attitudes scores, most of them were equal or lesser than 4.0. The low practices score may due to at least two main possible reasons. One was that the “practice” part was listed at the end of the questionnaire. Some respondents were unable to complete the questionnaire properly, resulting in some missing values. The other reason was the lack of right air pollution self-protection guidance. Perhaps people were unaware of what kind of measures should be taken to protect them from the polluted air. Also, data showed that parents’ protective ways were inappropriate in both of the districts. Taking all our results together, it can be clearly seen that air pollution education is important, not only to give residents the basic knowledge of the potential hazards of poor air quality and the correlation between air pollution and children’s respiratory diseases, but also to inform of the appropriate actions to take in order to protect themselves and their families. This is especially essential among those with lower educational levels or AAHI. In addition, many studies show that formal education increase mothers’ understanding of multiple dimensions of health and this comprehension of health translates into a greater use of maternal and child health service [37]. Based upon previous experience, various methods can be used to promote the air pollution education in China, such as: distributing education brochures, organizing regular education lectures, or free respiratory system screenings. Air quality and children’s respiratory health-related education campaigns are also one of the most effective approaches to enhance residents’ awareness of ways to monitor and improve air quality and children’s health [38]. However, long-term environmental health education programs are still needed to change the evolving situation. Meanwhile, the majority of the respondents (nearly 90%) from both settings agreed that improving the air quality is the responsibility of every citizen, which means most of the respondents had high awareness, and sense of obligation towards environmental protection. So strengthening air pollution education systems may be one of the most effective methods to involve all the citizens in air quality improvement. Our result is consistent with a previous study by Jacobi, which reported that residents felt that both the public and private sectors had an equal responsibility to improve the air quality [39]. On the other hand, compared to the parents from the community setting, respondents with ill children worried much more about their children’s health and believed that the government had not spent enough funds to improve air quality. Parents from the hospital group may be more likely to express concerns over these issues because they are more exposed to the detrimental effects of poor air quality with their ill children. 

The association between K-A, K-P and A-P were slightly positively correlated in the hospital, community and the whole of the respondents (Spearman’s rank correlation coefficient approximately 0.40). Knowledge of health behaviors considered to be beneficial, however, does not automatically mean that this behavior will be followed. The results obtained from this survey assessing the degree of knowledge are helpful to identify areas where information and education efforts should be strengthened in future. 

Attitudes are not directly observable as practices, thus it is a good idea to assess them. It is interesting to note that numerous studies have often shown low, and sometimes no connection, between attitudes and practices [40]. In this study, we did not explore the reason why attitudes dose not translate into practices, but this point needs further investigation and could be the subject of future research.

There are some limitations for this survey. Firstly, this survey was conducted at two suburb sites in Shanghai, and the design of the study has limited participation of residents from urban areas where air quality is particularly poor. Secondly, the survey participants were limited to caregivers of children aged 1–12 years old, mostly parents and grandparents. Residents who have no children aged 1–12 years old may have different attitudes. Thirdly, as the KAP survey contained little (or no) open-ended questions, the question style fails to reveal new problems and deepen the understanding of the situation. Lastly, there was limited number of respondents; more could be learned from assessing large volumes of affected people. Thus, application of these findings to other areas needs to be done with caution.

## 5. Conclusions

A large percentage of residents, especially those with higher education and income, are aware of the severity of China’s air pollution problem, and the correlation between air pollution and children’s respiratory diseases. Although the current air quality is pretty well received by majority of the respondents, there is a clearly significant difference between the two groups in their attitudes and practices. KAP scores between the hospital and community groups were statistically significant in difference with parents and caregivers from the hospital group displaying higher KAP scores. These results emphasize the importance of improving awareness associated with air quality among residents in Shanghai through public education and environmental protection campaigns.

Almost all of the respondents (90%) agreed that improving the air quality is the responsibility of every citizen. The joint action of governments and all citizens should be effectively utilized for an enhanced control the currently serious problem of air pollution in Shanghai. In addition, we suggest that more resources should be allocated towards educating citizens of the air pollution problem and providing them with appropriate practices to help lessen the detrimental effects on their families, particularly their children.
